# The high resource impact of reformatting requirements for scientific papers

**DOI:** 10.1371/journal.pone.0223976

**Published:** 2019-10-30

**Authors:** Yan Jiang, Robert Lerrigo, Anika Ullah, Muthu Alagappan, Steven M. Asch, Steven N. Goodman, Sidhartha R. Sinha

**Affiliations:** 1 Division of Gastroenterology and Hepatology, Department of Medicine, Stanford University School of Medicine, Stanford, CA, United States of America; 2 University of California San Diego, La Jolla, CA, United States of America; 3 Department of Internal Medicine, Beth Israel Deaconess Medical Center, Boston, MA, United States of America; 4 Division of Primary Care and Population Health, Department of Medicine, Stanford University School of Medicine, Stanford, CA, United States of America; 5 Center for Innovation to Implementation, VA Palo Alto, Menlo Park, CA, United States of America; 6 Division of Epidemiology, Department of Health Research and Policy, Stanford University, Stanford, CA, United States of America; Universita degli Studi di Ferrara, ITALY

## Abstract

**Background:**

Most research manuscripts are not accepted for publication on first submission. A major part of the resubmission process is reformatting to another journal’s specific requirements, a process separate from revising the scientific content. There has been little research to understand the magnitude of the burden imposed by the current resubmission process.

**Methods:**

We analyzed original research article submission requirements from twelve randomly selected journals in each of eight scientific and clinical focus areas from the InCites Journal Citation Reports database. From the 96 journals selected, we randomly identified three recently published manuscripts and sent surveys to those first and/or corresponding authors (288 total) to solicit information on time spent reformatting resubmissions and opinions on the process.

**Findings:**

There was significant variation in manuscript submission requirements for journals within the same scientific focus and only 4% of journals offered a fully format-free initial submission. Of 203 authors responding (71.5% response rate), only 11.8% expressed satisfaction with the resubmission process and 91% desired reforming the current system. Time spent on reformatting delays most publications by at least two weeks and by over three months in about 20% of manuscripts. The effort to comply with submission requirements has significant global economic burden, estimated at over $1.1 billion dollars annually when accounting for a research team’s time.

**Interpretation:**

We demonstrate that there is significant resource utilization associated with resubmitting manuscripts, heretofore not properly quantified. The vast majority of authors are not satisfied with the current process. Addressing these issues by reconciling reformatting requirements among journals or adopting a universal format-free initial submission policy would help resolve a major subject for the scientific research community and provide more efficient dissemination of findings.

## Introduction

The process of publishing peer-reviewed research can be slow and onerous [1–2]. It is not uncommon for manuscript reviews to take three months and the overall time from submission to publication to take between seven to nine months [3–5]. This process is filled with multiple potential sources of delays such as awaiting reviewer feedback and incorporating edits from multiple co-authors. Determining methods to increase value and minimize inefficiencies in research is an area of substantial interest [6–12]. Topics explored have included streamlining research protocols, making data more widely available, and reducing unnecessary bureaucracy to start projects. A focus has been made on identifying reasons for the potential delays in order to improve the overall efficiency of the research process [1, 13–14].

One obstacle for authors is the need to reformat a rejected manuscript when submitting to a new journal [15–17]. Valuable suggestions have been made regarding the general required sections of manuscripts [18–19]. While this certainly promotes some uniformity, it does not fully address the more granular and highly variable formatting prerequisites for manuscript publications that, even today, are common with publishers. With an average manuscript submission rejection rate of about 62%, much higher at top tier journals, this delay affects the majority of submissions and could have substantial cumulative effect [20]. This is particularly relevant as it seems there are some trends, at least for some of the most selective journals, which would further increase the delays and resources required to ultimately publish. The manuscript acceptance rate for *Nature*, for example, has decreased nearly 30% in the last 20 years [21]. These delays and additional efforts used towards publication of research can also lead to poor utilization of public and private funds, which are commonly dedicated to promote efforts that advance science. The inefficiency of this process also hampers career advancement as promotions are often time sensitive and directly related to the publication of research findings [1, 14, 22]. Journal editors have also shared a common author sentiment that time for revisions could be spent more productively on findings and data [23].

Despite this, there has been little research to understand the magnitude and reasons for resubmission delays. In this study, we investigate how much formatting variation exists amongst a diverse set of scientific journals. We then conduct a survey of recently published researchers to determine how much time is spent reformatting manuscripts before journal acceptance and the largest contributors to publication delay. Lastly, we evaluated the authors’ satisfaction with the current process and their recommendations for change.

## Materials and methods

### Ethical statement

Our research study was approved by the Stanford University institutional review board (#43750). The data sets that support the findings of this study are publically available at the Harvard Dataverse: https://doi.org/10.7910/DVN/B5HJQX.

### Journal selection

We randomly selected twelve journals from the InCites Journal Citation Reports (JCR) database in each of eight broad scientific (biology, biochemistry and molecular biology, microbiology, immunology, and cell biology) and clinical fields (cardiology, gastroenterology, oncology). The OpenEpi random number generator was used to select each numerically assigned journal from the InCites JCR website [24]. The basic and clinical sciences were chosen to encompass a wide range of primarily biomedical researchers. To be included in the study, the journal must have contained original research articles (as defined by each journal) and be either published in the United States or, in rare cases, have multiple US based editors. For each journal, we reviewed the instructions for authors and extracted key formatting requirements for publication including manuscript word limits, abstract word limits and structure requirements, maximum number of tables/figures, and citation limits. We also collected additional information on impact factor, cover letter requirements, and the number (if any) reviewers needed to be suggested. Upon completion of multiple reviews by members of the research team, we then contacted the editorial team (from online contact listings on each journal’s respective website) of journals included in this manuscript to verify the data we extracted and offer them an opportunity to comment on our assessments.

From each of the 96 journals selected, we randomly selected three recently published manuscripts. We again used a random number generator to pick each article, which was pre-designated a number. We picked articles starting from the most recent issue at that time of review (September—October 2017). The following inclusion criteria were used: 1) considered an original research article as defined by the journal, 2) publicly available contact information displayed for the first and/or corresponding authors and 3) the first author/corresponding author must be affiliated with a US-based institution. The latter criterion was required for communication purposes as regular mail was used to obtain a portion of surveys as described below.

### Survey administration

All 288 first authors from the 96 journals randomly selected were sent an email with a link to complete an eight-question survey on reformatting for journal resubmissions (supporting material, S1 Text). These questions largely solicited information on the time spent on reformatting resubmissions, the reformatting categories responsible for the time spent, and the authors’ opinions on the resubmission process. It was explicitly stated in the survey that any estimates of time spent on reformatting should be not include any effort spent improving the actual scientific content of the manuscript.

Two email reminders to complete the survey were sent at weekly intervals to authors who did not respond. If the first author failed to respond after three total emails, the same initial email and two weekly reminder emails, as needed, were then sent to the listed corresponding author. If no response was received after emails to both the first and corresponding author, we sent a mailed copy of the survey with pre-paid return postage to the corresponding author with a nominal monetary incentive of $5 to be kept if they sent the completed survey back. We requested the money be returned if the survey was not completed. We accepted mailed survey responses up to one month after mailing. Those who completed the survey, either online or by mail, were entered in a drawing to win a $100 Amazon.com gift card.

### Cost estimations of reformatting

**Fig 1:**
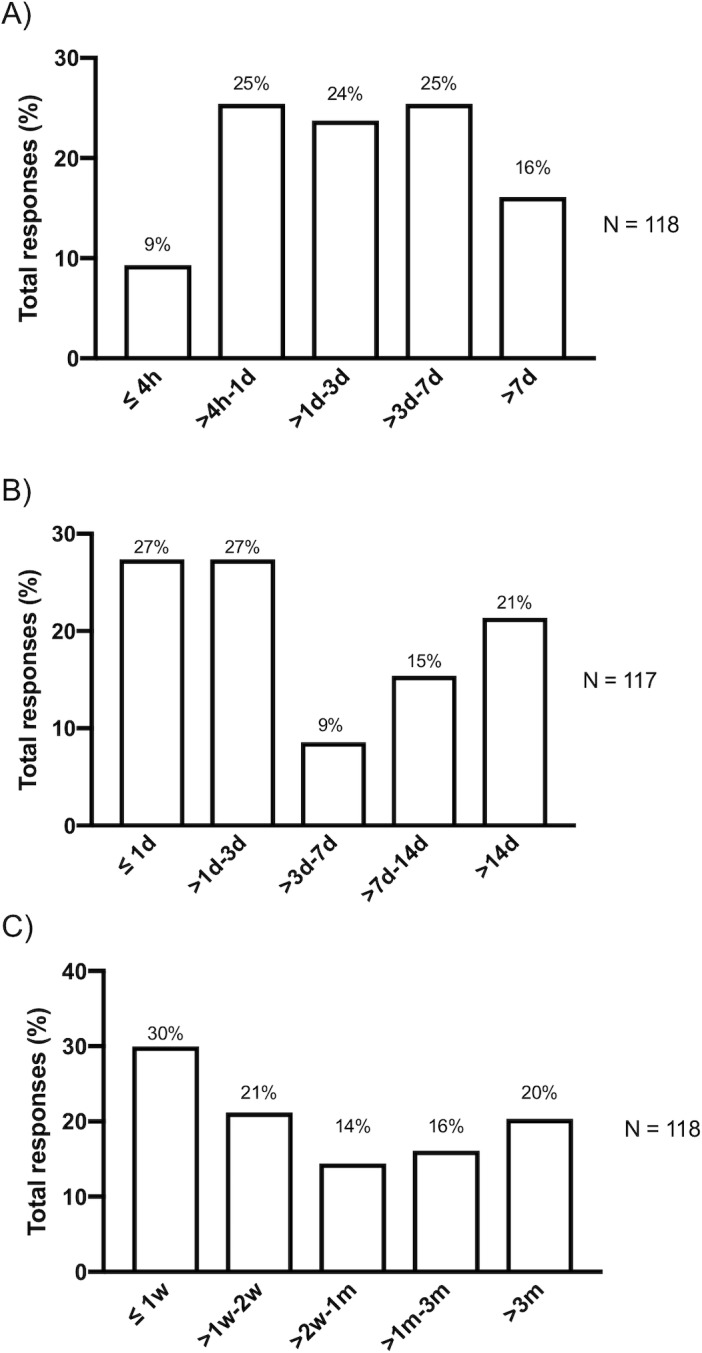
Distribution of time spent on reformatting: (A) by author (B) by research team. (C) Resubmission delay times caused by reformatting.

To provide a rough, “back-of-the-envelope” calculation of the economic burden from reformatting, we used NIH research stipend data for first year postdoctoral scholars. This salary, $48,432, is consistent with previously published postdoctoral salaries, when adjusted for inflation to 2018 [25–26]. To determine the number of publications per year, we examined multiple previously published sources, all with several inherent limitations. The National Science Foundation (NSF), for example, estimates about 2.3 million publications worldwide yearly, but this estimate does include some non-medical engineering publications [27]. Another approximation cited in the literature indicates roughly 2.5 million annual publications [20, 28]. Other calculations, based on SCOPUS (Elsevier’s abstract and citation database) and Web of Science (Clarivate Analytics citation indexing service), have concluded there are about 2.2 million yearly research publications [29]. For the sole purpose of illustrating that the cost of reformatting is not insignificant, we multiplied the number of publications (based on NSF data) by the percent of articles requiring resubmission based on our survey data results. We then multiplied this number by the hours spent reformatting from our survey data and the hourly rate of a postdoctoral scholar as described above in order to determine a rough estimate of cost due to reformatting. For the hours spent on reformatting, we used the lower limits of time ranges shown in Fig 1A (1 hr, 4 hrs, 1 day, 3 days, 7 days) and Fig 1B (1 hr, 1 day, 3 days, 7 days, 14 days).

### Statistical analysis

Survey questionnaire was administered and the data was collected using the Stanford REDcap database [30]. Organization of data and graphing was done using Excel (Microsoft, 2011) and Prism 8.0 (GraphPad, 2018). To analyze ordinal data amongst two groups, we used the Mann-Whitney test with significance set at a p level of 0.05 or less (two-tailed). Statistical testing was done using Prism 8.0 (GraphPad, 2018).

## Results

### Variation in journal requirements

There was significant disparity in manuscript requirements across all journal types. We compared 12 key variables for manuscript publication across all 96 journals selected for review. To illustrate the heterogeneity of these manuscript format requirements, the results for microbiology and gastroenterology journals are shown in Table 1 as examples.

**Table 1:** The heterogeneity of journal requirements in randomly selected gastroenterology and microbiology publications.

Journal Name	2016 JCR Impact Factor	Format-Neutral Initial Submission (Y/N)	Cover Letter Required (Y/N)	Author Contribution Section (Y/N)	# of Suggested Reviewers	Total Word Limit	Total Character Limit	Total Page Limit	Abstract Word Limit	Standard Abstract Structure? (Y/N)	Standard Body Structure (Y/N)	Max # Tables & Fig	Maximum # Citations
**Gastroenterology** **Journals**													
Pancreas	2.967	N	Y	N	N/A	N/A	N/A	N/A	200	Y	Y	N/A	N/A
Gastrointestinal Endoscopy	6.501	N	N	Y	N/A	3500	N/A	N/A	250	Y	Y	N/A	50
American Journal of Gastroenterology	9.566	N	N	N	N/A	N/A	N/A	N/A	250	Y	Y	N/A	N/A
Inflammatory Bowel Diseases	4.525	N	N	N	N/A	N/A	N/A	N/A	250	Y	Y	N/A	N/A
Journal of Clinical Gastroenterology	3.328	N	Y	N	N/A	N/A	N/A	N/A	250	Y	Y	N/A	N/A
Gut	16.658	N	Y	Y	Y but no specifics	4000	N/A	N/A	250	Y	Y	N/A	50
Hepatology	13.246	N	Y	N	Y but no specifics	6000	N/A	N/A	275	Y	Y	8	50
Liver Transplantation	3.91	N	Y	N	Y but no specifics	5000	N/A	N/A	275	Y	Y	N/A	50
Clinical and Translational Gastroenterology	3.923	N	Y	Y	2	N/A	N/A	N/A	250	Y	Y	N/A	N/A
Digestive Diseases and Sciences	2.875	N	Y	N	4 to 6	N/A	N/A	N/A	250	Y	Y	N/A	N/A
Journal of Viral Hepatitis	4.122	N	N	N	3	4000	N/A	N/A	250	Y	Y	6	N/A
Journal of Neurogastroenterology and Motility	2.457	N	Y	Y	N/A	N/A	N/A	N/A	250	Y	Y	N/A	N/A
**Microbiology** **Journals**													
Diagnostic Microbiology and Infectious Disease	2.401	N	N	N	2	3500	N/A	N/A	150	Y	Y	5	N/A
mBio	5.621	N	N	N	3	5000	N/A	N/A	250	N	Y	N/A	N/A
Journal of Bacteriology**	3.143	N	Y	N	3	N/A	N/A	N/A	250	N	Y	N/A	N/A
PLOS Pathogens	6.608	N	Y	Y	4	N/A	N/A	N/A	300	Y	Y	N/A	N/A
Clinical and Vaccine Immunology*	2.425	N	Y	N	N/A	5000	N/A	N/A	250	N	Y	N/A	N/A
ISME	9.664	N	Y	N	N/A	5000	N/A	N/A	200	N	Y	8	100
Environmental Microbiology	5.395	N	Y	N	5	N/A	N/A	10	200	N	Y	N/A	N/A
Cell Host & Microbe	14.946	N	Y	Y	3	N/A	55000	N/A	150	Y	N	7	N/A
FEMS Microbiology Ecology	3.720	N	Y	N	N/A	N/A	N/A	N/A	200	Y	Y	N/A	N/A
Molecular Microbiology	3.898	N	Y	Y	6	N/A	N/A	N/A	200	N	Y	N/A	N/A
Clinical Infectious Diseases	8.216	N	Y	N	4	3000	N/A	N/A	250	Y	N/A	N/A	40
Frontiers in Microbiology	4.076	N	Y	Y	N/A	12000	N/A	N/A	350	N	Y	15	N/A

n/a in sections where there is no specific mention of requirement

*Clinical and Vaccine Immunology has changed to mSphere journal since initial query. Data above is for Clinical and Vaccine Immunology.

**Journal of Bacteriology now offers format free publishing but not at time of initial query

Even among journals with the same scientific focus, the requirements for publication were highly variable. For example, within gastroenterology journals, 7/12 format dimensions showed heterogeneity. Only 4/12 journals explicitly required an author contribution section and 8/12 required a cover letter. Manuscript word limits were only mentioned for 5/12 journals (and varied from 3500 to 6000 words). A request for suggested reviewers was made in 6/12 journals, 2/12 journals had specific table/figure limitations, and 4/12 had restrictions on the number of citations accepted. Additionally, while all of the gastroenterology journals required an abstract, the word limits for the abstract varied from 150 to 300 words.

For microbiology journals, the heterogeneity was even greater. For example, an author contribution section was required in only 4/12 journals. Suggested reviewers were mentioned in 8/12 publications. Standard paper body structure was either not required or not mentioned in 2/12. Though a structured abstract was commonplace in gastroenterology journals, this was not the case for microbiology, as 7/12 journals did not require standard abstracts. At the time of our review, only 4/96 (4%) of journals offered fully format-free initial submission.

### Population sample

We received a total of 206/288 responses (71.5%) to our survey of United States based biomedical researchers (Table 2). Mail-in responses accounted for about 20% of our total (41/206). First authors responded most often (158/206, 76.7%). Authors who were listed as both first and corresponding were categorized as first authors. The majority of manuscripts in our study (N = 118, 57.3%) were not accepted by the first journal to which it was submitted. Subgroup analysis comparing basic science and clinical journals revealed no significant differences in these resubmission rates, so this data was not included in manuscript.

**Table 2:** Survey responder characteristics.

**Survey Responses (n, %)**	
Total	206/288 (71.5%)
Mail In	41/206 (19.9%)
First Author**	158/206 (76.7%)
Corresponding Author	48/206 (23.3%)
**Responses by journal type (n, %)**	
Clinical Science*	71/206 (34.5%)
Basic Science	135/206 (65.5%)
**Number of submissions before acceptance (n, %)**	
1	88/206 (42.7%)
2	55/206 (26.7%)
3	35/206 (17.0%)
4	18 /206 (8.7%)
5+	10 /206 (4.9%)

*Clinical Science defined as JCR identified journals in gastroenterology, cardiology or oncology

**If author listed as both first and corresponding, he/she was counted as first author

Of note, we received eighteen email bounce backs from the initial email survey request. For six authors, we were able to publicly search for a replacement email address that was up to date. For the other twelve, which we were not able to find an updated email address, we re-selected twelve new articles in the same respective journals and resent the survey request email to the twelve new first authors with no further bounce backs.

### Time spent on resubmission

The distribution of time spent reformatting by survey responder and by the entire research team is shown in Fig 1A and 1B. When asked how much time was needed for reformatting to all journals to which the paper was resubmitted to, the majority of authors (77/118, 65%; Fig 1A) reported that they spent 1–3 days or more (one day of effort was defined to the respondent as meaning eight hours). This did not include time spent on improving the scientific content or waiting for reviewer comments. Time spent on reformatting alone delayed resubmissions by over two weeks in most instances (60/118, 51%; Fig 1C). Though delay to publication did vary based on how many submissions were required before acceptance, 20% of submissions were postponed over three months due to format revisions alone.

**Fig 2:**
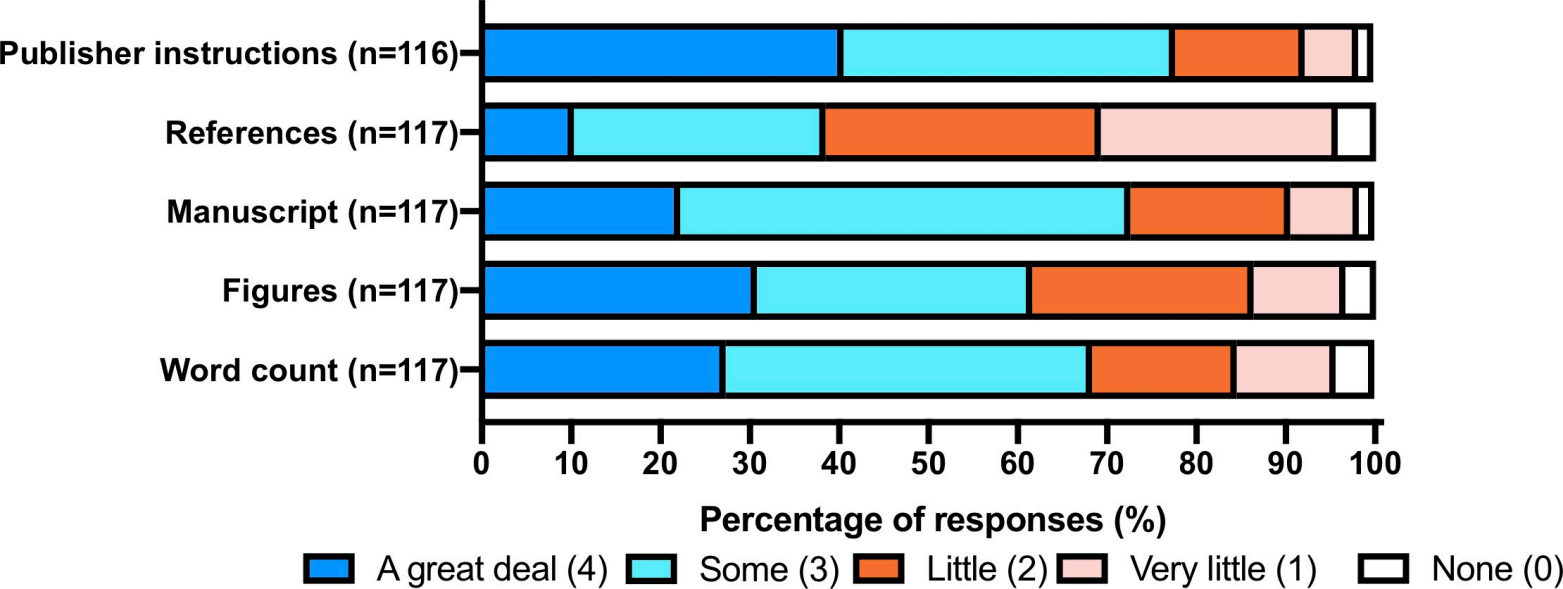
Author’s opinion on burden of time (0–4 scale) spent on various common reformatting issues.

Fig 2 shows the distribution of time spent by authors on common reformatting tasks such as word count, figures, references, manuscript structure, and publisher online submission requirements. Given the difficulty in quantifying exact hours on these tasks, authors were given options on a 0–4 effort scale ranging from none (0) to a great deal (4). These authors expressed that most time was spent on various journal/publisher online requirements (e.g., entering author contact information, disclosures, etc.) with 90/116 (78%) expressing that this took them some to a great deal of time. The only task that the majority of investigators felt was associated with minimal time expenditure (i.e. little, very little, or no time spent) was readjusting references (72/117, 62%).

### Views on current journal submission format

**Fig 3:**
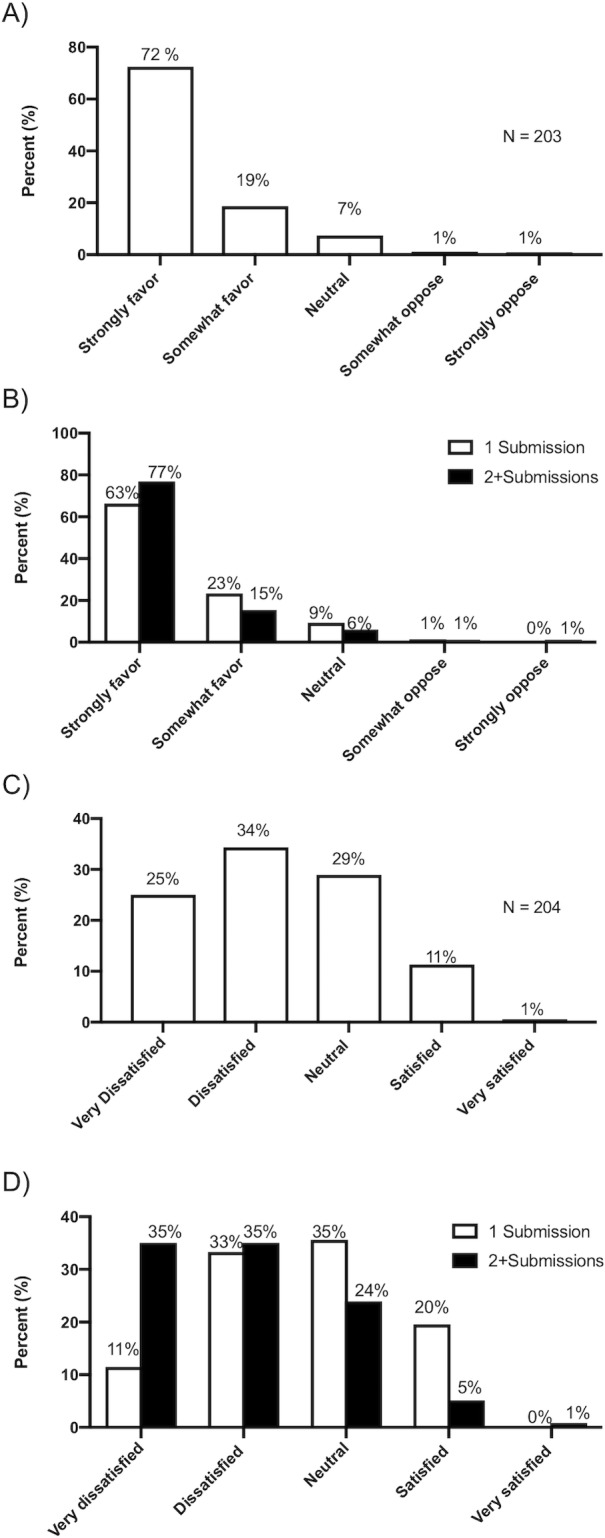
(A) Authors’ attitudes toward streamlining resubmission process. (B) Attitudes toward streamlining, 1 vs 2+ submissions. (C) Satisfaction with current system. (D) Satisfaction with current system,1 vs 2+ submissions.

Only 11.8% of 203 authors expressed satisfaction with the current resubmission process, with 59.3% reporting dissatisfaction (Fig 3C). 91.1% of authors favored a more streamlined approach to reformatting (Fig 3A).

Dissatisfaction varied by the number of submissions prior to acceptance (Fig 3D). Compared to those who only submitted to one journal, those who submitted to at least 2 reported greater scale of dissatisfaction (82/117 (70%) compared to 39/87 (45%), p<0.05). However, this did not affect the very high percentage who desired change (92% for multiple submissions vs. 90% for single submission, p = 0.11 (Fig 3B).

### Yearly estimates on time and costs related to reformatting

Based on our data of 57.3% of articles needing resubmission, the time spent on reformatting (Fig 1), and prior data of 2.3 million annual scientific articles published [27], we estimate that first or corresponding authors spend about 23.8 million hours reformatting worldwide every year. Using the average first year postdoctoral researcher salary of $48,432 [25–26], we roughly estimate costs of reformatting to be around $550 million dollars yearly worldwide for the first or corresponding author. When taking into account the time spent by the entire research team (Fig 1B), the costs are estimated to be $1.1 billion dollars. As the United States makes up nearly 18% of publications [27], reformatting costs when restricted to US based papers, are estimated to be about $202 million dollars annually.

## Discussion

We demonstrate that authors bear a substantial burden when revising manuscripts for journal resubmission. Among the time-consuming processes involved are adjusting manuscript structure (e.g. altering abstract formats), changing figure formats, and complying with word counts that vary significantly depending on the journal. Beyond revising the manuscript itself, authors often have to adjust to specific journal and publisher online requirements (such as re-inputting data for all authors’ email, office addresses, and disclosures). Most authors reported spending “a great deal” of time on this reformatting task. Reformatting for these types of requirements reportedly caused three month or more delay in the publication of nearly one fifth of articles and one to three month delays for over a third of articles.

The cumulative time burden to researchers is greater than previously hypothesized. Previous estimates on time spent (e.g. at least one hour per each rejected article) on reformatting were based on editorial and opinion pieces rather than actual representative data [20,22]. Our data show that nearly 91% of authors spend greater than four hours and 65% spend over eight hours on reformatting adjustments before publication (Fig 1A). A prior survey-based research study on biomedical journal publications times noted a median time of first submission to acceptance of five months but this seemingly included all delays in the publication process (including review time and changes to improving scientific content).

The research community has expressed frustration about the amount of time often highly trained scientists are devoting to reformatting instead of to discovery [15, 22]. According to a cited post by a managing editor, the formats are mainly there “to make their review and publication process run more smoothly” [23]. Others have pointed out that different formats exist for mainly stylistic purposes so journals have another distinguishing feature to make them stand out in a competitive publishing landscape [15–16].

For authors, such delays can increase training times, delay career advancement, and decrease chances of securing time-sensitive grants [2, 14]. These delays can have significant economic burden as well. We show that cost estimates of reformatting can be upwards of $1.1 billion dollars worldwide when accounting for time spent by an entire research team. There are several limitations in calculating this cost. The purpose, however, was to simply show, using a rough estimate, that the monetary cost of this issue is not trivial. There were several assumptions we had to make in order to come up with our estimates. First, the time spent on reformatting comes from our data, which is based on responses from only US based biomedical researchers. For salary, we chose a first year postdoctoral researcher salary, as we believed this to be the lowest labor cost of those involved in the research teams we sampled. We note that using salaries of post docs from other countries may reduce the cost estimate, but using salaries of more senior faculty, who are also involved in this reformatting, would increase these associated costs. For the number of articles published, there are several sources that have cited around 2.2–2.5 million yearly publications (as explained in our methods) and this estimate of about 2 million yearly publications has been published in recent work for similar estimates on time spent reformatting [20]. We opted to use the NSF estimate, as it is mostly limited to scientific publications. It did include non-original research articles, which our study did not address. It also included engineering articles, but we do not feel there is a strong reason to believe that reformatting issues related to that field would be dissimilar to the fields we sampled. We decided to provide worldwide cost estimates in this paper because we feel this issue is not just isolated to US based researchers—especially, as many publications will take manuscripts authored from non-US based scientists. Conservatively, however, we used the low end of time ranges spent on reformatting for all of these cost estimates; costs increase substantially when taking even the midpoint of the time ranges. These rough estimates make clear that the cumulative magnitude of this unnecessary hidden tax on the scientific community is not trivial.

Efforts to reduce these times expended in manuscript submission include increasing use of preprint servers such as bioRxiv and format-free initial submissions [16, 20, 23, 31]. Many of our respondents also offered suggestions as to how to make the reformatting process more efficient in the open response portion of our survey, which is included in our public dataset (see Methods section for link). The most common suggestions brought up by authors included adopting a common manuscript structure for prominent journals, the aforementioned format-free initial submissions, uniform reference formatting, and creating a central online author profile that could be exported into multiple publishers with pre-filled data (or linking to central repositories such as ORCID). One common theme expressed was a sense of urgency for change. Indeed, one explanation for the high response rate of 72% to our survey may be the degree of frustration amongst scientists with the current resubmission process. Given the high resource utilization of reformatting, additional avenues should be explored for improving the current process. For example, as research in the United States is often federally funded, the NIH could work with publishers to have a uniform initial submission format for work sponsored by taxpayer dollars.

Our study does have some important additional limitations. Inherent to a survey based study, there is risk for recall bias. By choosing the most recent publications, we hoped to limit this issue as much as possible. There is also possibility of voluntary response bias, particularly for those who have strong opinions on this matter (perhaps those whose papers required multiple re-submissions). A very robust response rate, however, and study data showing a high rate of authors (43%) having their manuscript accepted into their initial journal choice reduce the risk of this bias.

The majority of manuscripts are resubmitted, yet researcher’s efforts and opinions about reformatting requirements have not been adequately explored. We have found that researchers with recently published manuscripts are dissatisfied with the current resubmission process and expend substantial time on reformatting instead of on productive scientific activities. This is a potentially unnecessary burden with substantial collective scientific and financial impact. Our data supports reconciling reformatting requirements among journals and adopting a universal format-free initial submission policy.

## Supporting information

S1 Text Research survey sent to authors. (PDF)
