# Supplementary material for: The high resource impact of reformatting requirements for scientific papers
Source: PLoS One. 2019 Oct 30;14(10):e0223976. doi: 10.1371/journal.pone.0223976 (PMC6821399; doi:10.1371/journal.pone.0223976)
Supplement: S1 Text — (PDF) [file pone.0223976.s001.pdf]

Research Submission Inefficiencies

You are invited to participate in a Stanford IRB-approved research study examining the time and resources spent on preparing and submitting research manuscripts. The purpose of this research is to identify possible areas that could be improved in the manuscript preparation process that will potentially facilitate more timely dissemination of research. We do not anticipate any risks or direct benefits to participation in this survey. We cannot and do not guarantee or promise that you will receive any benefits from this study. You will not receive payment for your participation, but you will be entered into a drawing to win a $100 Amazon gift card if you complete the survey. If you have read this form and have decided to participate in this project, please understand your participation is voluntary and you have the right to withdraw your consent or discontinue participation at any time without penalty or loss of benefits to which you are otherwise entitled. The alternative is not to participate. You have the right to refuse to answer particular questions. Your individual privacy will be maintained in all published and written data resulting from the study.

Regarding your recently published article described in our email, please think of the time* you spent reformatting it for resubmission, if any. This reformatting is separate from any efforts spent improving the scientific content of your manuscript, and includes:

· Determining submission requirements for new journals
· Meeting new word counts, figure, and reference count requirements
· Changing the font, figure, and bibliography formats
· Uploading all submission information, such as all author contact information and cover letter
· Submitting and following up on author copyright, disclosure and role attestations

*For the purposes of the survey, one day refers to an 8 hour workday.

---

1. How many journals (including the accepting journal) did you submit this manuscript to before it was accepted?

◯ 1 (then skip to question 6)
◯ 2
◯ 3
◯ 4
◯ 5 or more

---

2. How much time did YOU personally spend reformatting (see definition above) the manuscript for submissions to all the journals to which it was resubmitted?

◯ less than or equal to 4 hrs,
◯ more than 4 hrs but less than or equal to 1 day
◯ more than 1 day but less than or equal to 3 days
◯ more than 3 days but less than or equal to 7 days
◯ more than 7 days

---

3. How much time did your RESEARCH TEAM (including you), administrative support staff, and coauthors together spend reformatting the manuscript for submission to all journals to which it was resubmitted? As a reminder, this is time spent on reformatting only, not on changes to the scientific content of your manuscript.

◯ less than or equal to 1 day
◯ more than 1 day but less than or equal to 3 days
◯ more than 3 days but less than or equal to 7 days
◯ more than 7 days but less than or equal to 14 days
◯ more than 14 days

---

4. In total, how many calendar weeks did reformatting delay the resubmission of your manuscript?

◯ less than or equal to 1 week
◯ more than 1 week but less than or equal to 2 weeks
◯ more than 2 weeks but less than or equal to 1 month
◯ more than 1 month but less than or equal to 3 months
◯ more than 3 months

Again, as you are completing the survey, please remember that these questions refer to reformatting, NOT on time spent improving the scientific content of your manuscript.

5. Please estimate how much time each of the following took:

Reformatting: word counts	◯ None<br>◯ Very little<br>◯ Little<br>◯ Some<br>◯ A great deal
Reformatting: figures	◯ None<br>◯ Very little<br>◯ Little<br>◯ Some<br>◯ A great deal
Reformatting: manuscript structure	◯ None<br>◯ Very little<br>◯ Little<br>◯ Some<br>◯ A great deal
Reformatting: bibliography	◯ None<br>◯ Very little<br>◯ Little<br>◯ Some<br>◯ A great deal
Completing online instructions, including entering authors' info and disclosures	◯ None<br>◯ Very little<br>◯ Little<br>◯ Some<br>◯ A great deal
Other	◯ None<br>◯ Very little<br>◯ Little<br>◯ Some<br>◯ A great deal
If you listed "other" in previous questions, please provide example of other reformatting issues.	__________________________________
6. How satisfied are you with the current process for reformatting resubmissions?	◯ Very Dissatisfied<br>◯ Dissatisfied<br>◯ Neutral<br>◯ Satisfied<br>◯ Very Satisfied
7. How much would you favor streamlining the reformatting process for submission of scientific manuscripts?	◯ Strongly oppose<br>◯ Somewhat oppose<br>◯ Neutral<br>◯ Somewhat favor<br>◯ Strongly favor
8. What recommendations do you have on how reformatting for journal submissions can be improved?	____
